# Rest versus exercise as treatment for patients with low back pain and Modic changes. a randomized controlled clinical trial

**DOI:** 10.1186/1741-7015-10-22

**Published:** 2012-02-29

**Authors:** Rikke K Jensen, Charlotte Leboeuf-Yde, Niels Wedderkopp, Joan S Sorensen, Claus Manniche

**Affiliations:** 1 Research Department, Spine Centre of Southern Denmark, Hospital Lillebaelt, Oestre Hougvej 55, 5500 Middelfart, Denmark; 2 Institute of Regional Health Services Research, University of Southern Denmark, Winsloewparken 19.3, 5000 Odense C, Denmark

## Abstract

**Background:**

Clinical experience suggests that many patients with Modic changes have relatively severe and persistent low back pain (LBP), which typically appears to be resistant to treatment. Exercise therapy is the recommended treatment for chronic LBP, however, due to their underlying pathology, Modic changes might be a diagnostic subgroup that does not benefit from exercise. The objective of this study was to compare the current state-of-the art treatment approach (exercise and staying active) with a new approach (load reduction and daily rest) for people with Modic changes using a randomized controlled trial design.

**Methods:**

Participants were patients from an outpatient clinic with persistent LBP and Modic changes. They were allocated using minimization to either rest therapy for 10 weeks with a recommendation to rest for two hours daily and the option of using a flexible lumbar belt or exercise therapy once a week for 10 weeks. Follow-up was at 10 weeks after recruitment and 52 weeks after intervention and the clinical outcome measures were pain, disability, general health and global assessment, supplemented by weekly information on low back problems and sick leave measured by short text message (SMS) tracking.

**Results:**

In total, 100 patients were included in the study. Data on 87 patients at 10 weeks and 96 patients at one-year follow-up were available and were used in the intention-to-treat analysis. No statistically significant differences were found between the two intervention groups on any outcome.

**Conclusions:**

No differences were found between the two treatment approaches, 'rest and reduced load' and 'exercise and staying active', in patients with persistent LBP and Modic changes.

**Trial Registration:**

ClinicalTrials.gov: NCT00454792

## Background

Exercise therapy is currently the recommended first-line treatment in clinical guidelines for chronic non-specific low back pain (LBP) [1]. However, a meta-analysis of exercise therapy trials in patients with chronic LBP has shown that despite statistically significant improvements, the effect size of exercise (mean treatment effect) was small for pain (7 points out of 100) and disability (3 points out of 100) compared with no treatment or other conservative treatments [2].

Clinical experience suggests that patients with chronic non-specific LBP can respond very differently to the same treatment. The reasons for this are unknown, as few studies have been able to identify predictors of a positive outcome [3]. However, one factor that may influence an individual's response to exercise treatment is the underlying etiology of the pain. In the case of chronic non-specific LBP, it is not unreasonable to expect such pain to be caused by a number of conditions, some of them involving identifiable pathoanatomical changes. If the same type of treatment were to be provided for a wide range of different conditions or pathologies, one might expect the outcomes to be dissimilar. If and how researchers should deal with subpopulations of LBP has attracted attention in recent years [4] and preliminary results suggest that targeting treatment for specific LBP subgroups might be more effective than generic treatments directed towards mixed populations with non-specific LBP [5].

Recently, focus has been put on a diagnostic subgroup of LBP, those with Modic changes (MCs). MCs can only be visualized using Magnetic Resonance Imaging (MRI) and have been described as being a stage of the disc degeneration process [6]. Modic *et al. *divided MCs into three types: Type I, II and III [7,8]. Histological studies show that Type I consists of fissured endplates and vascular granulation tissue adjacent to the endplate whereas Type II is characterized by a disruption of the endplates and fatty degeneration of the adjacent bone marrow [7]. Type III seems to be similar to sclerosis of the bone marrow as seen on plain-film radiographs [8].

A review of the prevalence of MCs and the association with LBP estimated the median prevalence of MCs in clinical populations at 43% and showed that MCs are less common in non-clinical populations, with a median prevalence of 6% [9]. Positive associations between MCs and LBP were found with odds ratios (ORs) ranging from 2 to 20 [9]. More recently, three additional studies [10-12] reported similar findings, in which ORs for the association were 5, 9 and 28. Although believed to be associated with disc degeneration, the precise etiology of MCs is unknown. One theory is that MCs are caused by mechanical stress [13]. It has been shown that disc degeneration alters the biomechanics of the disc [6]. Excessive loading and shear forces may result in microfractures of the endplate causing inflammation in the vertebral endplate and the adjacent bone marrow [14]. An association has been found between MCs and severely degenerated discs [15,16], and also with previous disc herniation [17].

Our clinical experience suggests that many patients with MCs have relatively severe and persistent LBP, which is commonly unresponsive to treatment. Furthermore, a retrospective study (an unpublished Masters thesis) at the Spine Center of Southern Denmark, showed that patients with MCs were less likely to improve with physical activity compared with patients with non-specific LBP [18]. One hypothesis is that patients with MCs treated with exercise are unlikely to improve because vigorous weight-bearing exercise might inhibit microfracture healing. This hypothesis is based on knowledge of treating microfractures in other parts of the body, for example in stress fractures in the lower extremity [19,20]. The extension of this hypothesis is that patients with LBP and MCs constitute a specific subgroup, which should not be treated with physical activity. It would be clinically useful to know if this were true, because the gold standard treatment for patients with persistent or chronic non-specific LBP is back exercise and encouragement to keep physically active [1,21].

Therefore, we undertook a controlled trial of patients with both MCs and persistent LBP. The objective of this study was to compare the current 'state-of-the art' treatment approach (exercise and encouragement to keep active) with a new approach (load reduction and daily rest). The underlying hypothesis for this new treatment approach was that if vertebral bone microfractures were present in MCs, the provision of sufficient time and rest to facilitate bone healing would improve patient outcomes.

## Methods

### Trial design

A two-group randomized controlled clinical trial was undertaken.

### Participants

Participants in this study were patients with persistent LBP who attended a specialized outpatient spine clinic (Spine Center of Southern Denmark) after referral from the primary care sector. Criteria for referral were: (1) back problems with or without radiculopathy, (2) a maximum of two years' duration of the current episode, and (3) previous appropriate but unsuccessful treatment in the primary care sector.

In this clinical setting, from August 2007 to December 2008, MRI was routinely performed [22] on all patients (with no contraindications for MRI) meeting the following criteria: (1) LBP or leg pain of at least 3 on an 11-point Numerical Rating Scale, (2) duration of current symptoms from 2 to 12 months, and (3) age above 18 years. These criteria were chosen on the basis of previous projects where there was a high prevalence of MCs.

All patients with an MRI showing MCs Type I, II or III with a distribution exceeding the endplate, were clinically examined by a researcher (RKJ) to determine if they met the inclusion criteria. The researcher was blinded to subsequent patient allocation to intervention groups. Eligible patients were then informed about the results of the MRI, showing that MCs were a possible reason for their LBP and invited to participate in the trial. As directed by the Ethics Committee for the Region of Southern Denmark, both written and oral information about the trial was provided to each patient before admission to the project. This information also contained a layperson's explanation of the rationale for both treatment approaches. A team consisting of a nurse, a medical practitioner, a physiotherapist and a chiropractor attended participants during the trial if any complications occurred or if any pain medication required modification.

### Inclusion and exclusion criteria

Patient inclusion criteria were: (1) LBP of at least 3 on an 11-point Numerical Rating Scale, (2) LBP greater than any leg pain present, (3) duration of current symptoms from 2 to 12 months, (4) age from 18 to 60 years, (5) ability to read and speak Danish, (6) a willingness to participate in the study, and (7) a minimum of one MC (Type I, II or III) that extended beyond the endplate into the vertebral body.

Patients were excluded if they: (1) were unable to participate in the project because of other physical or mental disorders, (2) had a competing LBP etiology such as disc herniation with symptomatic root compression, or (3) had undergone previous spinal surgery with no pain relief after the operation.

### MRI evaluation

MRI was performed with a 0.2 T MRI-system (Magnetom Open Viva; Siemens AG, Erlangen, Germany). A body spine surface coil was used with the patient in the supine position. The imaging protocol consisted of sagittal and axial T1- and T2-weighted sequences.

The MRI evaluation, at baseline, was performed by a musculoskeletal radiologist (JSS) with extensive MRI experience and trained in using the standardized evaluation protocols [23,24] used in this study. MCs were evaluated according to the Nordic Modic Consensus Group classification [23], which had shown substantial to almost perfect reproducibility with Kappa values for intra-observer reproducibility (k = 0.77 to 1.0) and inter-observer reproducibility (k = 0.73 to 0.91).

**Figure 1:**
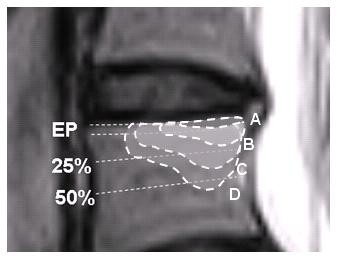
**Classification of size**. Classification of the size of a Modic change (MC) based on its depth of extension into the vertebral body height: **A**: Endplate only, **B**: > endplate to 25%, **C**: 25 to 50%, and **D**: > 50%. Only patients with B, C or D met this inclusion criterion for the current study.

Patients were included if the 'maximum height' of the MC extended beyond the endplate into the vertebral body on the sagittal image (Figure 1). This criterion was chosen to enhance the probability that this MRI finding was clinically relevant and because it had been shown to have high Kappa values for intra-observer reproducibility (k = 0.83) and inter-observer reproducibility (k = 0.80) [23].

### Interventions

Patients wishing to participate in the project were allocated to one of two groups, the new treatment approach (rest group) or a comparison treatment (exercise group).

The rest group was instructed to avoid hard physical activity and to rest twice daily for one hour, by lying down. To imitate the session structure in the exercise group and thereby the potential effect of being in a group [25], patients met in a 'café-like' environment which provided the opportunity to exchange personal experiences of pain and physical incapacity. They met in groups of up to a maximum of 10 people once every second week for 10 weeks for a session of 45 minutes where they engaged in unstructured talk with a physiotherapist present. To remind the patients not to be physically active and to attempt to support the spine, they were given an orthopedic flexible lumbar belt from 'Camp Scandinavia' and were instructed to use it as needed for up to a maximum of four hours per day. Patients were instructed to use a diary to record separately how many hours a day they were resting and wearing the lumbar belt. After 10 weeks, patients were instructed to increase their physical activity gradually until a self-determined acceptable level for the patient was achieved.

The exercise group received exercises for the stabilizing muscles in the low back and abdomen together with dynamic exercises, exercises for postural instability and light physical fitness training. The patients exercised in groups of up to a maximum of 10 people for one hour once a week for 10 weeks guided by a physiotherapist. They were encouraged to do the same exercises at home three times a week and to maintain a 'normal' level of activity. The patients had the opportunity to socially interact with each other during the sessions. After 10 weeks of intervention, the patients were given directions on how to continue their exercise at home. This intervention represented the normal 'active' treatment as recommended in current clinical guidelines [1,26].

Two physiotherapists were assigned to each of the two intervention groups for the duration of the trial, although only one attended each session. These four physiotherapists had 12, 15, 20 and 37 years of experience, respectively, working with back pain patients. For both interventions, two nurses, one for each group, participated in two of the sessions to discuss pain medication, introduce pain coping strategies and to tackle 'unhelpful' beliefs about LBP. The first session in both groups started with 20 minutes of information about anatomy and MCs as well as repetition of the rationale for treating MCs with either exercise or rest. Only the rationale that was relevant for the group to which the patient was allocated was explained.

The patients' attendance was recorded at each session and if they were absent, they were contacted by a secretary to determine the reason for their non-attendance and to help to remove any obstacles to participation. When the patient had more than 20% absence, she or he was counted as a dropout.

A detailed protocol of procedures and content of the sessions was made for each intervention group for use by the attending nurses and physiotherapists, to ensure a uniform and consistent approach to the interventions. There was no overlap of personnel between the two intervention groups. The interventions ran from August 2007 to April 2009 with an intake of patients every fifth week to ensure that patients waited a maximum of five weeks from their initial examination to commencement of their involvement in the intervention.

### Variables

A booklet of questionnaires was distributed to patients to complete in their own time at baseline, 10 weeks from baseline (post-treatment) and 12 months from the end of intervention (one-year follow-up).

### Variables collected at baseline

#### MRI

Information on the presence and type of MCs for 11 endplates from upper L1 to upper S1 was extracted using the evaluation protocol (Nordic Modic Consensus Group classification [23]).

#### Questionnaires

Questionnaire information was collected at baseline (before allocation) containing the following background variables: age, sex, body mass index (BMI), smoking status, employment status, type of occupation, education level, whether currently on sick leave and days on sick leave during the previous year. The outcome measures of pain, disability and general health described below were also contained in the baseline questionnaire booklet. In addition, information on any ongoing complaint at the National Social Appeals Board, the Patient Complaints Board of the National Health Service or a financial compensation case was collected together with information on any previous treatment administered in relation to the present episode of LBP. Finally, patients were asked how they would expect the treatment approaches (rest and exercise, respectively) to affect their back pain. A five-point Likert scale for each treatment with response options ranging from 1 (much better) to 5 (much worse) was used as measurement.

### Variables collected post-treatment (10 weeks) and at one-year follow-up

Details on the primary and secondary outcome measures are described in Additional File 1.

### Primary outcome measure

#### Pain

The numerical rating scale [27] (NRS) measures current back pain on a 0 to10 scale.

### Secondary outcome measures

#### Disability

The Roland Morris Disability Questionnaire [28] (RMQ) is a 23-item disability questionnaire with a 0 to 23 scale, measuring activity limitation.

#### Generic Health

EuroQol [29,30] (EQ-5D) is a standardized instrument measuring health status-related quality of life consisting of a health status index (EQ_index _0 to 1 scale) and a visual analogue scale (EQ_VAS _0 to 100 scale).

#### Global assessment

The global assessment transition questionnaire measures the patients' perceptions of the overall change in their back pain since the beginning of the study on a 7-point Likert scale [31].

#### Depression

Beck Depression Inventory [32] (BDI) is a 21-question inventory measuring the presence and severity of depressed mood with a 0 to 63 scale.

#### SMS-Track (pain and sick leave)

In order to obtain detailed information about each patient's clinical course, automated text messages via short message service tracking (SMS-Track) [33] were used for data collection. SMS-Track is a system for data capture. Each week, for 52 weeks starting at baseline, a programmed database sent a text message to patients' cell phones containing two questions: 'How many days have you had low back problems during the last week?' and 'How many days have you been on sick leave due to your back problem during the last week?' Patients were instructed to answer from '0' to '7' according to the number of days per week that were relevant for each question. If the patient did not answer the question within five days, a text message reminder was automatically sent. If the patient had three or more missing answers, a secretary called the patient and asked if the patient had any problems with the technique and if so, tried to solve the problem. The sick leave data were transformed into a five-day week instead of a seven-day week, by recoding answers with six or seven days as five days.

### Additional information

Post-treatment and at the one-year follow-up, any adverse events associated with the treatments, together with a description of any care-seeking for the current back problem (that is, having consulted a general practitioner (GP), specialist, chiropractor, physiotherapist, other care provider or been hospitalised) was also measured.

### Sample size

A power calculation was made using a data file (unpublished observations) from the Spine Center of Southern Denmark, which showed that patients with MCs on a numerical rating scale (0 to 10) reported an average pain of 6.4 (SD 1.8). The aim was to have 90% power to observe a significant difference (alpha level) of 5%. To ensure a mean difference of 30% or more between the groups and with an estimated dropout rate of 20%, we needed to include 38 patients in each group.

### Randomization

On receiving the completed baseline questionnaire, the project secretary allocated each patient into one of the two intervention groups by means of computerized minimization software ('Minim', an MS-DOS program [34]). Minimization [35] is a covariate adaptive randomization method [36] which aims to ensure that treatment arms are balanced with respect to certain predefined factors as well as for the number of patients in each group [37]. The technique has been shown to be a highly effective allocation method recommended in the conduct of controlled trials particularly when trial sample sizes are small [37]. Variables equally distributed through minimization were age (3 groups), sex (2 groups), heavy smoking, that is, ≥ 20 cigarettes a day (2 groups) and self-reported hard physical work (2 groups). The variables were chosen for the following reasons: age because activity level and care-seeking might be influenced by age [38], sex because women more often report some kind of consequences due to spinal problems than men [38], workload and smoking because hard physical work in combination with heavy smoking is associated with MCs [39]. A concern about using minimization is the risk of selection bias due to the potential for assignment being predicted if the person conducting the minimization has knowledge of the characteristics of earlier patients [37]. This was managed by ensuring that minimization was consecutive in order of patient registration and by the task of minimization being shared between two people. The researchers were masked to group assignment.

### Blinding

The MRI evaluator (JSS) was blinded to all patient information except name, sex and age. This was achieved by sending the images to an external radiologist with no access to other study data. The researchers analyzing the data (RKJ and NW) were blinded to patient identification as the study participants were given a random identification number when assigned to the project. All researchers were blinded to identification of the groups until all the analyses were done. The key for identification of patients and group was concealed by the project secretary.

### Data management and analysis

Comparisons were made of baseline data between the two intervention groups, and between compliers and non-compliers in each group using t-tests or a non-parametric equivalent for continuous variables and a chi-square test or Fisher exact test for categorical variables.

All data were analyzed using the intention-to-treat (ITT) principle, whereby all patients who returned a questionnaire were included regardless of their participation in the intervention. In addition, an efficacy subset analysis was carried out for the primary and secondary outcome measures, selecting the subset of patients who received their treatment and who did not drop out for any reason.

For the whole study sample, the mean difference between baseline and each of the two follow-ups for primary and secondary outcome measures were compared using paired t-tests. A comparison between groups was performed using multiple linear regression with robust variance estimations. Regression models were adjusted for baseline score and for the following covariates: age, sex, hard physical work and heavy smoking. The covariates were included because it has been suggested that factors used for minimization in the allocation process should be adjusted for in the analyses [37].

The number of patients in each group who achieved a minimal clinically important difference (MCID) was counted for the pain and disability outcomes using the raw change score from baseline to follow-up. A potential group difference was analyzed using a chi-square test. MCID values for secondary sector patients from a study by Lauridsen *et al. *[40] were used because these MCID values were derived for patients undergoing standard treatment in the same clinical setting as the current study. The MCID values were 'quartile-specific', which means that the value of MCID is dependent on the quarter of the scale in which the baseline score is located. For the pain scale (0 to 10), the MCID is 1 if the baseline score lies in the first quarter of the scale (0 to 2.5), 2 if it lies in the second quarter (> 2.5 to 5), 4 if it lies in the third quarter (> 5 to 7.5), and 1 if it lies in the fourth quarter (> 7.5 to 10). For the RMQ scale (0 to 23), the MCID is 4 if the baseline score is in the first quarter of the scale (0 to 5.75) and 2 for the second and third quarter (> 5.75 to 17.25). No quartile-specific value was available for the fourth quarter, and so an overall score with the value 2 was used [40].

Items on the 7-point global assessment transition questionnaire were dichotomized into not better (that is, reports of 'much worse', 'worse', 'a little worse', 'about the same' and 'a little better') and better (that is, 'better' and 'much better') and the proportion of patients who improved in each group was compared using a chi-square test.

When analyzing the SMS-Track data, only patients with a total response rate of more than 80% were included. Generalized Estimating Equations (GEE) were used to assess any difference between the groups over time with age, sex, hard physical work and heavy smoking as covariates. Further, we counted the total number of days with back problems over the 52 weeks as a proportion of the maximum number of days possible. The same analysis was performed for the number of days on sick leave, assuming each working week consisted of 5 days. The groups were compared using a chi-square test. The SMS-Track data were also presented visually. Adverse events and care-seeking were reported in raw numbers and percentages.

Finally, an analysis was performed investigating if the patients' expectations of the treatment they received would influence the outcome [41]. The five-point Likert scale for each treatment option was dichotomized into not better (that is, reports of 'much worse', 'worse' or 'unchanged'), and better (that is, 'better' and 'much better'). A dichotomous 'treatment expectation' variable was constructed containing patients who received their preferred treatment and those who did not. A comparison of the distribution in the two intervention groups was analyzed using Fisher's exact test. Thereafter, a regression model was created with intervention group and the 'treatment expectation' variable as the independent variables, and the dependent variables (outcome variables) of pain, disability and general health. The model was adjusted for baseline score, age, sex, hard physical work and heavy smoking.

Significance (alpha level) was set at 5%. All analyses were performed using the Stata statistical software, version 11.

### Approval

The study was approved by the Ethics Committee for the Region of Southern Denmark, approval # S-VF-20060111 and registered in ClinicalTrials.gov Identifier # NCT00454792.

## Results

### Participant flow

**Figure 2:**
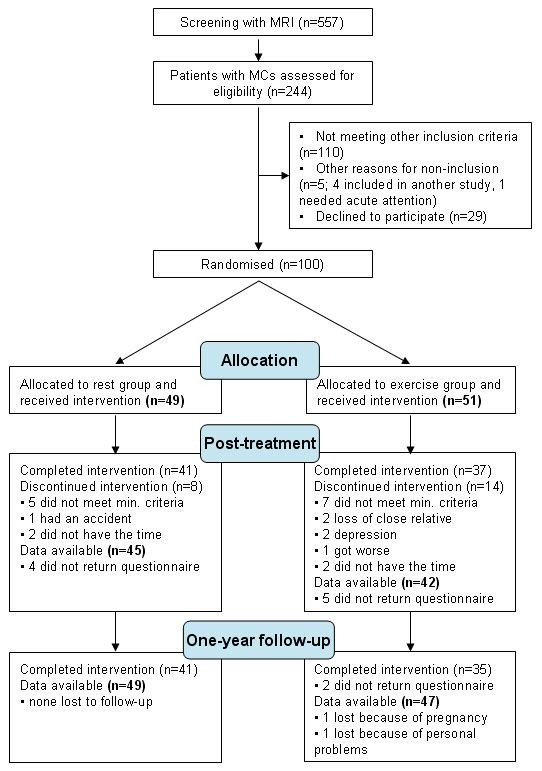
**Flowchart**. Flow of patients referred to the project and included in the interventions, together with an overview of dropouts and the reasons for this.

A flow diagram summarizing the study process, including drop-out rates and reasons for drop-out, is illustrated in Figure 2. In total, 557 patients were screened with MRI and, of those, 244 (44%) had MCs with a 'maximum height' extending beyond the endplate into the vertebral body. Of the 244 patients with MCs who were potential participants, 100 were included in the interventions, and 144 did not meet inclusion criteria (Figure 2). Data on 87 patients post-treatment, and 96 patients at one-year follow-up were available and used in the data analysis (ITT).

### Compliance

At the end of the intervention period, 78 patients had completed the full treatment program (dropout rate 22%). Of those who completed the intervention, two patients declined participation in the one-year follow-up. In total, 78 patients post-treatment and 76 patients at one-year follow-up had completed the intervention. These participants were used in the efficacy analyses.

On average, patients participated in 84% of the sessions in the rest group or 91% of sessions if only those who completed the intervention were considered. In the exercise group, patients participated in 74% of the sessions or 88% of sessions if only completers were considered. Patients in the rest group rested on average for 1 hour and 34 minutes per day (range 18 to 161 minutes) and they used the lumbar belt on average for 1 hour and 33 minutes per day (range 0 to 291 minutes). Those who completed the intervention rested on average 1 hour and 40 minutes per day (range 56 to 161 minutes) and used the belt on average for 1 hour and 34 minutes per day (range 1 to 291 minutes).

### Outcomes and estimation

#### Baseline data

Table 1 shows baseline demographic and clinical characteristics for the whole study sample and for each intervention group. There were no significant differences between the two groups regarding any of the baseline variables, including patient expectations of treatment effect. Patients who dropped out during the intervention did not differ significantly on any baseline variables compared with patients who completed the intervention.

**Table 1:** Baseline characteristics of participants in each of the intervention groups

Characteristic	Rest	Exercise	All
Number enrolled	49	51	100
Age [mean, SD]	47 ± 9.8	45 ± 8.9	46 ± 9.3
Sex [female %]	33 (67)	35 (69)	68 (68)
BMI [median, IQR]	25 (22-27)	25 (24-29)	25 (23-28)
BMI distribution [N (%)]			
Underweight < 18.5	0	0	0
Normal range 18.5 to 24.9	25 (51)	24 (47)	49 (49)
Overweight 25 to 30	16 (33)	17 (33)	33 (33)
Obese > 30	8 (16)	10 (20)	18 (18)
Smoking (Yes (%))	23 (47)	18 (35)	41 (41)
Heavy smoking (> 20 cigarettes/day) (N (%))	3 (6)	3 (6)	6 (6)
Employed (Yes (%))	34 (69)	39 (76)	73 (73)
Unemployed (Yes (%))	7 (15)	6 (12)	13 (13)
Disability pension (Yes (%))	4 (9)	2 (4)	6 (6)
Applied disability pension (Yes (%))	3 (6)	3 (6)	6 (6)
Type of occupation (N (%))			
Sitting	11 (22)	6 (12)	17 (17)
Mostly walking	17 (35)	23 (45)	40 (40)
Walking and some lifting	11 (22)	12 (24)	23 (23)
Hard physical work	10 (20)	10 (20)	20 (20)
Education (Number (%))			
Basic school 8 to 10 grade	17 (35)	14 (28)	31 (31)
High school	2 (4)	2 (4)	4 (4)
Vocational education	20 (41)	21 (41)	41 (41)
Academic, maximum 4 years	10 (20)	12 (25)	22 (22)
Academic, > 4 years	0 (0)	2 (4)	2 (2)
Sick leave (Yes (%))	18 (37)	20 (39)	38 (38)
Sick leave last year Number (%))			
0 days	8 (16)	9 (18)	17 (17)
1 to 30 days	17 (35)	19 (37)	36 (36)
31 to 365 days	24 (48)	23 (45)	47 (47)
Complaint or compensation case (Yes (%))	5 (10)	3 (6)	8 (8)
Pre-trial exercise therapy (Number (%))	30 (61)	23 (45)	53 (53)
MCs Type I (%)	38 (78)	40 (78)	78 (78)
MCs Type II (%)	30 (61)	31 (61)	61 (61)
MCs Type III (%)	9 (18)	4 (8)	13 (13)
Pain NRS (0 to 10) (mean ± SD)	5.6 ± 1.5	5.1 ± 2.2	5.3 ± 1.9
Disability RMQ-23 (mean ± SD)	12.0 ± 4.0	13.3 ± 4.8	12.6 ± 4.4
General health EQ_index (_mean ± SD)	0.68 ± 0.12	0.62 ± 0.18	0.65 ± 0.15
General health EQ_VAS (_mean ± SD)	54 ± 18	53 ± 20	53 ± 19
Depression BDI	10.7 ± 6.1	9.6 ± 5.9	10.2 ± 6.0

BDI, Beck Depression Inventory; BMI, body mass index; EQ-5D, EuroQol; IQR, interquartile range; NRS, numeric rating scale; RMQ, Roland Morris Disability Questionnaire; SD, standard deviation.

#### Follow-up data

Overall, the efficacy subset analyses on the outcome measures of pain, disability, general health and global assessment did not produce results that were different from the results of the ITT analyses and therefore only the results of the ITT analyses will be reported.

#### Primary and secondary outcomes

There were no differences between the two intervention groups for any of the outcomes of pain, disability, general health, depression, global assessment or the numbers of patients achieving an MCID. Estimates are summarized in Table 2 and Table 3. The pre- and post-treatment BDI variables were found not to follow a normal distribution, and therefore a square root transformation was performed, which effectively corrected the skew. Table 2 reports the untransformed scores to assist with clinical interpretation and the results from both transformed and untransformed analysis for comparison.

**Table 2:** Mean scores for pain, disability, general health and depression from baseline to follow-up and adjusted change scores

Outcome	Group	N	Mean (± SD)	Adjusted difference^a^ (95% CI)	*P*
**Pain NRS (0-10)**					
Baseline	Rest	49	5.6 (± 1.5)		
	Exercise	51	5.1 (± 2.2)		
Post-treatment	Rest	45	5.0 (± 1.9)	-0.07 (-0.9 to 0.7)	0.9
	Exercise	42	4.5 (± 2.1)		
One-year	Rest	48	4.8 (± 2.3)	-0.3 (-1.3 to 0.6)	0.5
	Exercise	46	4.3 (± 2.4)		
**Disability RMQ-23**					
Baseline	Rest	49	12.0 (± 4.0)		
	Exercise	51	13.3 (± 4.8)		
Post-treatment	Rest	45	11.0 (± 4.8)	-0.6 (-2.2 to 1.0)	0.5
	Exercise	42	11.1 (± 5.4)		
One-year	Rest	47	10.7 (± 5.5)	-1.2 (-3.3 to 1.0)	0.3
	Exercise	46	10.7 (± 6.1)		
**General health EQ_index_**					
Baseline	Rest	47	0.7 (± 0.12)		
	Exercise	51	0.6 (± 0.17)		
Post-treatment	Rest	43	0.7 (± 0.12)	0.04 (-0.007 to 0.09)	0.1
	Exercise	42	0.7 (± 0.13)		
One-year	Rest	48	0.7 (± 0.21)	0.06 (-0.008 to 0.14)	0.08
	Exercise	47	0.7 (± 0.13)		
**General health EQ_VAS_**					
Baseline	Rest	48	54 (± 18)		
	Exercise	49	53 (± 20)		
Post-treatment	Rest	44	56 (± 18)	0.02 (-7.7 to 7.7)	0.9
	Exercise	41	56 (± 21)		
One-year	Rest	49	56 (± 21)	5.4 (-2.6 to 13.5)	0.2
	Exercise	47	60 (± 22)		
**Depression score BDI**					
Baseline	Rest	49	10.7(± 6.1)		
	Exercise	51	9.6(± 5.9)		
Post-treatment	Rest	45	8.6(± 6.1)	0.67 (-0.99 to 2.3)	0.4
	Exercise	42	7.9(± 5.5)	*0.08 (-0.3 to 0.4)*^b^	*0.7*^b^
One-year follow-up	Rest	49	9.5(± 7.1)	-0.92 (-2.8 to 0.97)	0.3
	Exercise	47	8.0(± 6.1)	*-0.17 (-0.6 to 0.22)*^b^	*0.4*^b^

^a^Between group differences adjusted for baseline score, age, sex, physical workload and smoking. ^b^Results of analysis with square-root transformation of data. Mean values reported for untransformed data to assist clinical interpretation.BDI, Beck Depression Inventory; CI, confidence interval; EQ-5D, EuroQol; NRS, numeric rating scale; RMQ, Roland Morris Disability Questionnaire; SD, standard deviation.

**Table 3:** The number of patients who achieved a minimal clinically important difference (MCID) in pain and disability at 10-weeks (post-treatment), at one-year follow-up and at both time-points

	**Post-treatment**			**One-year follow-up**			**Both**
			
	**Rest**	**Exercise**	**All**	**Rest**	**Exercise**	**All**	**All**
	
Pain	9 (20%)	4 (10%)	13 (15%)	4 (8%)	9 (20%)	13 (14%)	2
Disability	4 (9%)	5 (12%)	9 (10%)	4 (9%)	7 (3%)	7 (8%)	1

**Figure 3:**
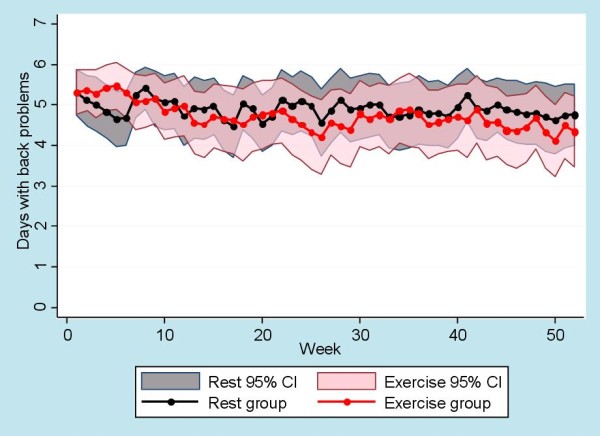
**SMS-Track of low back problems**. SMS-Track data showing means and CI for number of days with low back problems for both groups. The CI of the rest group is colored grey and is visible behind the transparent pink CI of the exercise group.

**Figure 4:**
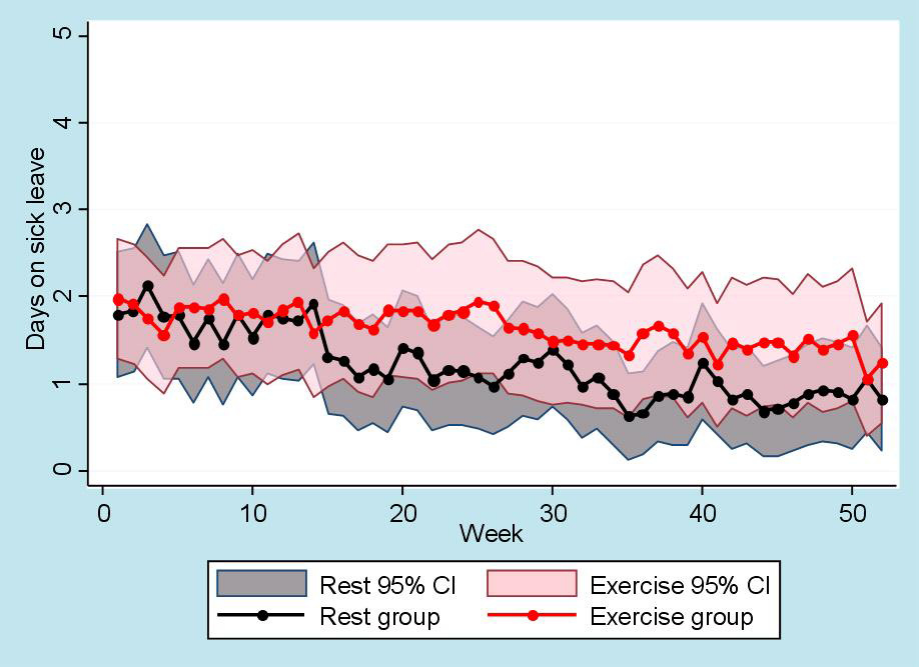
**SMS-Track of days on sick leave**. SMS-Track data showing means and CI for number of days on sick leave for both groups. The CI of the rest group is colored grey and is visible behind the transparent pink CI of the exercise group.

Seventy-nine patients had an SMS response rate of more than 80% and the following results are reported for those patients regardless of intervention drop-out status. The regression analysis of the SMS-Track data showed no significant difference between intervention groups for either LBP problems or sick leave. The total number of days with LBP problems was 70% of the maximum possible days for the rest group and 68% for the exercise group with no significant difference between the groups. The total number of days on sick leave was 24% of the total possible days for the rest group and 33% for the exercise group with no significant difference between the groups. For visual interpretation of the SMS-Track data, see Figure 3 for days with low back problems and Figure 4 for days on sick leave.

### Additional information

#### Additional treatment for LBP

Post-treatment, 8 patients (18%) in the rest group and 10 (26%) in the exercise group had sought additional consultations or treatments from external care providers for their current back problem. At one-year follow-up, the proportion of patients seeking additional care had risen to 30 (64%) in the rest group and 23 (50%) in the exercise group. This difference was not statistically significant at either time-point (See Table 4).

**Table 4:** The number of patients who had sought additional consultations or treatments from external care providers for their current back problem at 10-weeks (post-treatment) and at one-year follow-up

	**Post-treatment**			**One-year follow-up**		
		
	**Rest**	**Exercise**	**All**	**Rest**	**Exercise**	**All**
	
GP	4 (9%)	5 (13%)	9 (11%)	15 (32%)	14 (30%)	29 (31%)
Specialist doctor	0 (0%)	2 (5%)	2 (2%)	6 (13%)	5 (11%)	11 (12%)
Chiropractor	2 (5%)	5 (13%)	7 (8%)	12 (26%)	11 (24%)	23 (25%)
Physiotherapist	2 (5%)	1 (3%)	3 (4%)	14 (30%)	10 (22%)	24 (26%)
Other	4 (9%)	1 (3%)	5 (6%)	9 (19%)	2 (4%)	11 (12%)

GP, general practitioner.

#### Reported adverse events

No serious problems were reported post-treatment but in the rest group, two patients reported that they found it stressful to find the time to rest two hours a day, three reported increased pain and two reported pain from wearing the lumbar belt. In the training group, five patients complained about increasing pain after training sessions and one reported one episode of peripheralization of the pain. At one-year follow-up, there were no reported adverse events from the interventions.

#### Expectations

The distribution of patients who received the treatment of which they had positive expectations was equally distributed between the intervention groups (67% in the rest group and 75% in the exercise group, *P *= 0.4). Receiving the intervention that a patient had positive expectations of was not associated with outcome in pain, disability or general health compared with those who received a treatment they did not expect to be effective.

#### Secondary observations

When the whole study sample was considered, a statistically significant but small improvement was found in most of the outcome measures (See Table 5). The global assessment showed that post-treatment, 12 patients (14%) rated themselves 'better' or 'much better' and at one-year follow-up, this was 23 patients (24%).

**Table 5:** Mean change of outcomes for the whole study sample

Outcome	Follow-up	Mean difference *(95% CI)*	*P*
**Pain**	Post-treatment	0.6 (0.2 to 1.0)	0.004
**NRS (0-10)**	One-year	0.8 (0.3 to 1.3)	0.003
**Disability**	Post-treatment	1.5 (0.7 to 2.2)	0.000
**RMQ-23**	One-year	2.2 (1.1 to 3.2)	0.000
**General health**	Post-treatment	-0.04 (-0.07 to -0.005)	0.02
**EQ_index_**	One-year	-0.03 (-0.08 to -0.01)	0.18
**General health**	Post-treatment	-4 (-8.7 to 0.7)	0.09
**EQ_VAS_**	One-year	-5 (-9.4 to -0.5)	0.03
**Depression**	Post-treatment	3.0 (1.8 to 11.3)	0.000
**BDI**	One-year	1.7 (0.8 to 2.7)	0.000

Mean differences, confidence interval (CI) and *P*-values for outcome measures at baseline, post-treatment and at one-year follow-up. BDI, Beck Depression Inventory; EQ-5D, EuroQol; NRS, numeric rating scale; RMQ, Roland Morris Disability Questionnaire.

Compliance with rest and use of the lumbar belt varied considerably. It is plausible that patients with a high compliance would have a better outcome. However, a post-hoc analysis (data not shown) indicated that those who rested the most (top 30%) were no different in outcome for pain and disability than those with poorer compliance in the rest group.

## Discussion

### Main findings

There was no statistically significant difference between the two treatment approaches for any outcome measure at any of the time-points.

### Methodological considerations

The study might have been strengthened by the inclusion of a control group consisting of a 'no treatment' group. However, as participants were all seeking care for their LBP and as reviews and international guidelines recommend exercise as effective first-line treatment for chronic LBP[1,21], exercise therapy was used as the comparative treatment.

All patients were referred from primary care where treatment, including exercise, had been attempted in 53% of the patients. It is reasonable to assume that the exercise treatment had been ineffective since the patients were referred for secondary care. It is possible that this could have negatively affected the patients' expectations of exercise treatment, however this was not reflected in the analysis concerning the patients' expectations of treatment.

### Reflections on the new treatment approach (rest group)

In this study, the novel intervention being investigated consisted of a combination of rest and a lumbar belt. Evidence does not endorse this approach for non-specific LBP but we were targeting this treatment to a pathoanatomic-specific subgroup based on a biologically plausible hypothesis about the etiology of the pain.

Some physicians use MCs as an indication for prescribing lumbar belts as treatment for LBP [42] but the rest approach has not been investigated previously for people with both LBP and MCs and therefore, the effectiveness of this treatment could not be anticipated. Although a recent Cochrane review reported that 'it is still unclear if lumbar supports are more effective than no or other interventions for the treatment of LBP' [43], we were unable to find studies investigating the clinical effect of using a lumbar belt specifically as treatment for MCs.

The duration of the intervention for the rest group was selected to allow time for microfracture healing [44] and to match the duration of the exercise intervention. The number of hours that the patients were advised to rest per day was arbitrary but it was designed to allow participants to maintain normal work hours and to preclude the necessity for sick leave.

### Reflections on the comparison treatment (exercise group)

A previous study of patients with non-specific LBP at our centre compared physical training with education [45] and had patient selection criteria similar to the current study. The physical training group had a 25% improvement in pain and an 8% improvement in disability at one-year follow-up. In the current study, the exercise group improved 16% for pain and 21% for disability and, therefore, the improvement follows a similar trend to that reported by others in a comparable study population.

There is no compelling evidence that any particular type of exercise is more effective than another for the treatment of chronic LBP [46]. To expedite the study, the content of the exercise intervention was similar to that already provided at the Spine Center of Southern Denmark. One guideline recommends that the duration of an exercise program be a minimum of 12 weeks [26] but for logistical reasons we were constrained to 10 weeks, which might have been a limitation.

The exercise group in the current study had supervised sessions only once a week. Members were encouraged to do home exercises three times a week in which they had been instructed. Compliance with home exercise is generally considered to be poor [47], and supervision by a therapist is therefore recommended [46]. Compliance with performing the exercise program at home was not measured and the dosage unknown. The low frequency of supervised exercise sessions (once a week) is not optimal and could have influenced the effectiveness of the exercise therapy.

The individual preference of the physiotherapists and nurses involved in the intervention was unmeasured but may have influenced the outcome of the treatment. Both intervention approaches had face validity and the rationale for the study was communicated to all members of the study team. Blinding the therapists from the study hypothesis was not realistic and could potentially create bias, as the personal 'belief' in one rationale over another could have been projected onto the participants. However, the therapists involved had had substantial experience, as multiple studies have been conducted at the Spine Center previously and they were aware of the risk.

### Results and hypothesis

The absence of a between-group difference in this study challenges its underlying assumptions and hypothesis. Firstly, we had assumed that MCs were the cause of pain in this patient cohort. However, it is likely that spine-related pathoanatomical changes other than MCs or perhaps psychosocial factors may have influenced or caused the pain. Secondly, if MCs were the main cause of pain in this cohort and if rest were useful, the dosage may have been insufficient. It is possible that a few hours of rest a day is not sufficient to counteract the load induced by the activities of daily life. Thirdly, MCs consist of histologically different subtypes and it may be that subgroups of type, size and location of MCs could respond differently to treatment. Such a subanalysis of predictive factors for outcome will be reported in another paper. Lastly, the underlying hypothesis that MCs are caused by a biomechanical alteration of the disco-vertebral complex could be inadequate. Other hypotheses of explanatory pathoanatomic pathways exist, such as the possibility that the inflammation seen on MRI in MCs is caused by low virulent bacteria [13].

There was a small yet statistically significant improvement at both time-points for pain and disability at a whole group level. However, because we did not include a 'no treatment' group, we were not able to test whether the slight improvement occurring in both groups was due to an equal treatment effect in both groups or if the treatments had no effect and the improvement was a consequence of the natural course. Also, as this study sample consisted of patients with MCs only, we do not know if the overall poor prognosis is unique for this subpopulation. To explore this would require a different study design including patients both with and without MCs.

### Implications

By introducing the rest approach there may have been a risk of maintaining unwanted behaviors such as poor coping strategies, kinesiophobia, anxiety and catastrophization as studies have indicated that exercise treatment reduces some of these behaviors [48,49]. The potential psychosocial implications of the new approach might not have been sufficiently measured in this study, but we did not find a difference in emotional functioning (measured with BDI) between the groups. Although exercise was not superior to the rest approach in the current study, the concept of exercise and keeping active might have more general beneficial effects for physical and mental health. In addition, exercise is a well established treatment for chronic LBP and the results of this single study are not sufficient to recommended rest as an equal alternative for patients with MCs. However, based on our findings, it is reasonable to consider the possibility that not all subgroups of patients with persistent non-specific LBP will benefit to the same extent from exercise. Furthermore, when the relatively small effect sizes of conservative treatment (including exercise) for non-specific LBP in general are taken into account, we recommend that the resource consumption of treatment programs should be considered as a secondary outcome in future research. In addition, we recommend more research in the area of identifying subgroups that could more effectively benefit from the treatment provided.

## Conclusions

There was no statistically significant difference on any outcome measure between the treatment approach of rest and reduced load and the conventional approach of exercise and staying active.

## Abbreviations

BDI: Beck Depression Inventory; BMI: body mass index; CI: confidence interval; EQ-5D: EuroQol; GEE: generalised estimating equations; GP: general practitioner; ITT: intention to treat; k: kappa value; LBP: low back pain; MC: Modic change; MCID: minimal clinically important difference; MRI: magnetic resonance imaging; NRS: numerical rating scale; OR: odds ratio; RMQ: Roland Morris Disability Questionnaire; SD: standard deviation; SMS: short message service.

## Competing interests

The authors declare that they have no competing interests.

## Authors' contributions

RKJ participated in conception and design, carried out the data collection and the analyses, and wrote the main parts of the manuscript. CLY, NW and CM participated in conception and design and made substantial contributions to the manuscript. JSS made a substantial contribution to the acquisition of data and revising the manuscript. All authors have given their final approval of the version to be published.

## Pre-publication history

The pre-publication history for this paper can be accessed here:

http://www.biomedcentral.com/1741-7015/10/22/prepub

## Supplementary Material

Additional file 1 **Details on the primary and secondary outcome measures**. Click here for file
