# Supplementary material for: Rest versus exercise as treatment for patients with low back pain and Modic changes. a randomized controlled clinical trial
Source: BMC Med. 2012 Feb 29;10:22. doi: 10.1186/1741-7015-10-22 (PMC3348080; doi:10.1186/1741-7015-10-22)
Supplement: Additional file 1 — Details on the primary and secondary outcome measures. [file 1741-7015-10-22-S1.PDF]

Additional file 1

Outcome measure and description	Scale
**Numerical rating scale** (NRS)<br>A measure of current back pain intensity scaled from 0 representing no pain to 10 representing worst pain possible	0-10
**Roland Morris Disability Questionnaire** (RMQ)<br>A 23-item disability questionnaire developed specifically to measure activity limitation in LBP patients and is a modification of the original 24-item version. In this study the 23-item version was used because it has been validated in Danish. RMQ consists of 23 yes/no questions with the final score ranging from 0 (no disability) to 23 (extremely severe disability).	0-23
**EuroQol** (EQ-5D)<br>A standardised instrument measuring health status related quality of life consisting of a health status index (EQ_{index}) and a visual analogue scale (EQ_{VAS})	
EQ_{index}<br>Consists of five dimensions (mobility, self-care, usual activities, pain/discomfort and anxiety/depression) each of which can be scored with one of three responses. The responses allow for three levels of severity (no problems/some or moderate problems/extreme problems). The health status index is converted into a single summary index by applying a formula that essentially attaches values (or weights) to each of the levels in each dimension based on a value set based on the health status of the Danish general population. This gives a number ranging from 0 (death) to 1 (perfect health).	0-1
EQ_{VAS}<br>Self-rated health on a vertical, visual analogue scale where the endpoints are labelled ‘Best imaginable health state’ (100) and ‘Worst imaginable health state’ (0).	0-100
**Beck Depression Inventory** (BDI)<br>A 21-question self-report inventory measuring the presence and severity of depressed mood. The questions are scored on a scale value of 0 to 3 with a final score ranging from 0 to 63. The cut-offs are: 0–13: minimal depression; 14–19: mild depression; 20–28: moderate depression; and 29–63: severe depression.	0-63
**Global assessment**<br>A measure of patients' perception of the overall change in their back pain since the beginning of the study. This retrospective assessment was measured on a 7-point Likert scale ranging from “much better” to “much worse” with “unchanged” in the middle	
